# Systems Neuroscience’s 2022 Top Papers: An Editorial Summary

**DOI:** 10.3390/brainsci14040315

**Published:** 2024-03-27

**Authors:** Konstantin V. Slavin

**Affiliations:** 1 Department of Neurosurgery, University of Illinois at Chicago, Chicago, IL 60612, USA; kslavin@uic.edu; 2 Neurology Service, Jesse Brown Veterans Administration Hospital, Chicago, IL 60612, USA

Despite the seemingly endless—and sometimes overwhelming—flow of scientific information, there are always some articles that stand out from the crowd, either due to the depth of the covered topic, or due to their unique and unexpected findings. In the “Systems Neuroscience” journal section, both submissions and publications cover the widest spectrum of subjects, and each paper is, to some extent, a remarkable accomplishment of its own kind. Nevertheless, when asked to select top 10 papers of the calendar year, we have decide to use the most relevant indicator of importance—the number of citations—and the summary below covers exactly that: the top cited papers of 2022, following the tradition that we started last year [1].

Among the top ten papers from the “Systems Neuroscience” section are three reviews and seven original studies [2,3,4,5,6,7,8,9,10,11]. They cover a multitude of topics, ranging from neuromodulation to tinnitus to obesity and overweight status, and come from all over the world, with authors from Europe, Asia, North and South America, and Oceania. Each of them is definitely worth reading, and I would encourage the journal audience to consider at least glancing through their abstracts in search of useful information from near and far neuroscience topics.

In the first of the three papers on tinnitus in this list, Hall et al. summarized results of a fascinating clinical trial that was completed 10 years ago in two centers in the UK [2]. The authors recruited 100 patients with tinnitus (out of 391 screened), and randomized them into two equal, matched groups in order to determine the efficacy of desynchronizing pathological brain activity using a portable acoustic device. The patients were using the device with either a proprietary sound sequence or a placebo algorithm and, following 12 weeks of active double-blind comparison, an open-label extension tracked the results for another 24 weeks of therapy. I remember when the protocol of this study was published in 2013, the concept of “acoustic coordinated reset” was quite appealing and intriguing. The study did not find evidence of significant therapeutic benefit of the tested stimulation modality, and made the authors suggest alternative study designs for future investigations.

Tinnitus was also the subject of a different paper from the top 10 list: Cuesta et al. from Madrid analyzed the effects of an enriched acoustic environment with hearing-loss matched broadband noise as a part of combined treatment for patients with tinnitus [3]. Out of a cohort of 133 tinnitus subjects, they included 119 in as study participants, of whom 83 completed the treatment protocol consisting of initial counselling and a 4-month-long course of sound therapy. The sound stimulus used for daily hour-long sessions was personalized based on the individual audiometry data. In total, 80 of 83 patients completing the study attained clinically relevant improvement after the treatment was over, and the Tinnitus Handicap Inventory decreased by 23 (14–42) points, correlating the degree of improvement with severity of the condition. Despite the high attrition rate (36/119), the authors concluded that the efficacy of conventional treatment of tinnitus—the tinnitus retraining therapy—may be improved by designing an enriched acoustic environment (customized sound stimuli that are matched to the hearing loss curves of the patients).

To underscore the impact of chronic tinnitus on the patients’ lives, a group of German researchers analyzed results of a large questionnaire battery completed by a giant cohort of tinnitus patients (1958 patients!) over a 5 year period [4]. Brueggemann et al. aimed to determine which psychological aspects predict tinnitus-related distress and identify the underlying constructs through factor analysis. Based on this extensive dataset, which included sociodemographic data, tinnitus-related and general stress experiences, and emotional and somatic complaints, the authors concluded that both psychological and sociodemographic indices predict tinnitus-related distress, and a multitude of factors, including depressive exhaustion, somatization, general stress, reduced activity, and higher age, are relevant to the tinnitus-related distress existence.

To determine whether brain plasticity in patients with mild cognitive impairment (MCI) is affected by aerobic exercises, Farhani et al. conducted a systematic literature review focusing on randomized controlled trials [5]. To no one’s surprise, the authors of the review from Iran, Canada, and Japan came up with a total of only twelve relevant studies, some of which were focused on brain-derived neurotrophic factors, volumetric changes in brain structures, and brain activity measured with event-related potentials. There were no negative results among published studies and, although most showed an improvement with an exercise (elevation in trophic factors, increase in hippocampal volume, and positive changes in brain activity), some showed no changes among MCI patients. Based on this, the authors concluded that “aerobic exercise seems to be a promising therapy in people with MCI”, and its use is both cost-effective and associated with physical—and potentially cognitive—benefits.

A very interesting subject was investigated by Li et al. from Montreal: the authors analyzed 14 adult patients who were considered failures of epilepsy surgery (with the recurrence of seizures after their initial operation), and who were determined to have an insular epileptogenic focus [6]. The study was based on the surgical epilepsy practices of two well-established epilepsy centers (Montreal Neurological Institute [MNI] and University of Montreal Health Center [CHUM]). The insular epileptic activity was discovered after the surgical failure with intracranial EEG, magnetoencephalogram (MEG), or seizure improvement after subsequent insular resection. In retrospect, half of these patients (7/14) presented with manifestations suggestive of insular involvement before their first surgery—prompting the authors to suggest lowering the threshold for suspecting insular epilepsy during surgical planning. Out of the fourteen analyzed patients, nine underwent re-operations with insular resection, and seven achieved very favorable post-operative outcomes.

Wolf et al. reviewed the last 45 years of published literature on the subject of hypnosis, focusing on functional changes in brain activity due to hypnosis, as documented by objective testing [7]. The authors from several European countries came up with a total of 40 papers, and were able to identify evidence of hypnosis-induced functional changes based on electrophysiological (EEG, evoked potentials, EMG), neuromodulation (transcranial magnetic stimulation) and imaging (functional MRI, PET, SPECT, etc.) data. The heterogeneity of the studies made it difficult to generalize the results but, among many different investigated aspects, there were indicators of higher activity over the frontal brain areas and lower activity in the anterior cingulate cortex and the insula during hypnosis.

Two studies from the top 10 list dealt with non-invasive neuromodulation and the variability of its effects in response to changes in stimulation parameters. In the first of the two, a group of investigators from Japan analyzed the effects and after-effects of transcranial alternating current stimulation (tACS) delivered at different frequencies on brain oscillations and cortical excitability. Suzuki et al. first determined the optimal position of tACS electrodes through MRI-based simulation, and then tested the after-effects of tACS frequency in 16 healthy volunteers [8]. tACS delivered in alpha (10 Hz) and beta (20 Hz) frequencies resulted in larger alpha and beta oscillations, respectively. Moreover, tACS at these frequencies decreased the amplitudes of conditioned motor evoked potentials and increased alpha and beta activity, respectively, while enhancing cortical inhibition at the same time. These findings prompted the authors to conclude that tACS frequency differentially affects motor cortex oscillation and inhibition, and that cortical inhibition may change according to the tACS frequency-modulated balance between alpha and beta oscillations.

The second study on this general subject investigated non-invasive vagus nerve stimulation delivered through the left earlobe. The ability of the transcutaneous vagus nerve stimulation (tVNS) to affect the autonomic nervous system is well known, but the exact relation between the nature and scale of autonomic response and tVNS parameters remains unclear. To elucidate this topic, a group of researchers from Niigata, Japan, investigated the effects of different tVNS frequencies and current intensities on ECG measurements in a group of 35 healthy adult volunteers. Yokota et al. analyzed the changes in heart rate and other ECG parameters, including spectral analysis and heart rate variability, in a series of experiments while applying tVNS to the left earlobe [9]. Their findings confirm the current hypothesis of existence of sex differences in the degree of autonomic responses, and further suggest that both the stimulation parameters and sex difference should be taken into account when determining an optimal dose of stimulation in clinical applications of tVNS.

The recent literature on neuroplastic changes after brain stimulation has been thoroughly reviewed by Kricheldorff et al. [10]. The German researchers focused on three brain stimulation modalities (transcranial magnetic stimulation [TMS], transcranial electric stimulation [tES] and deep brain stimulation [DBS]), and limited their review to human studies applying neurostimulation. In an extensive narrative review, they presented the details of all three stimulation techniques, and summarized the evidence of neuroplastic capacity of each of them. Citing more than 250 references, the authors put together a deep dive into the current literature data on this important topic, but noted that some of the results conflict with each other, likely due to the small samples of the studies.

The last, but not least, on this list is the paper by La Marra et al. from Italy, presenting data on the executive functions of overweight and obese treatment-seeking patients [11]. The authors attempted to verify whether poor executive functions are related to a higher body weight, and whether executive functioning may contribute to weight loss in this patient cohort. The multitude of psychological and cognitive markers was compared between a cohort of 104 overweight and obese patients and a control group of 48 normal-weight subjects. Although obese patients obtained lower scores than overweight and normal-weight subjects in almost all executive measures, this between-group difference disappeared when sociodemographic variables were entered as covariates. These findings allowed the authors to conclude that there is evidence for the lack of association between obesity and the executive domains. The same group of authors published several other papers on this topic since the current study came out, and the most recent paper, also published in our journal in 2022 (but not included in the top 10 list), suggested that morbidly obese patients report lower performance on executive scores than obese, overweight, and normal-weight patients, and morbid obesity is associated with lower executive performance, even when sociodemographic covariates are taken into account [12].
